# Triketone-Modified Lignin and the Corresponding Biomass-Based Diketoenamine Resins: Synthesis and Properties

**DOI:** 10.1021/acsomega.5c07915

**Published:** 2026-01-19

**Authors:** Nien-Hsun Wu, Yi-Ming Sun, Ying-Ling Liu

**Affiliations:** † Department of Chemical Engineering, 34881 National Tsing Hua University, No. 101, Sec. 2, Kuang-Fu Road, Hsinchu 300044, Taiwan; ‡ Department of Chemical Engineering and Materials Science, 34895 Yuan Ze University, Chungli, Taoyuan 320315, Taiwan

## Abstract

This work reports the preparation of environmentally benign polymeric materials by employing biomass-based raw materials in the synthesis of recyclable thermosetting resins. With water-soluble lignin (possessing −COOH groups) as a feedstock, lignin-triketone (OL-TK) is prepared and cross-linked with tris­(2-aminoethyl)­amine (TREN) to result in the corresponding lignin-based diketoenamine resins. The resin shows a glass transition temperature (*T*_*g*_) of about 165 °C, which is higher than the values reported for other diketoenamine resins and other lignin-based vitrimers due to the relatively rigid structure of lignin and diketoenamine groups. Nevertheless, the lignin-based diketoenamine resin shows high brittleness, leading to failure in the mechanical tests. Further studies employ OL-TK as a reactive modifier for a furanic dicarboxylic acid (FDCA)-based diketoenamine resin. The lignin-containing diketoenamine resins exhibit some features of vitrimers, including satisfactory stress relaxation at 180 to 220 °C, thermal recycling ability, and chemical degradation/recycling characteristics. The addition of 3 wt % OL-TK brings positive effects on enhancing the mechanical properties and suppressing the creep at high temperatures of the pristine resin and does not alter its thermal properties and thermal recycling and reprocessing features. A high OL-TK fraction of 7 wt %, although further increasing the mechanical property and stress relaxation of the vitrimers, would result in lignin aggregation in the thermally recycled sample. High OL-TK content is negative for the recycling performance of the vitrimers.

## Graphical abstract

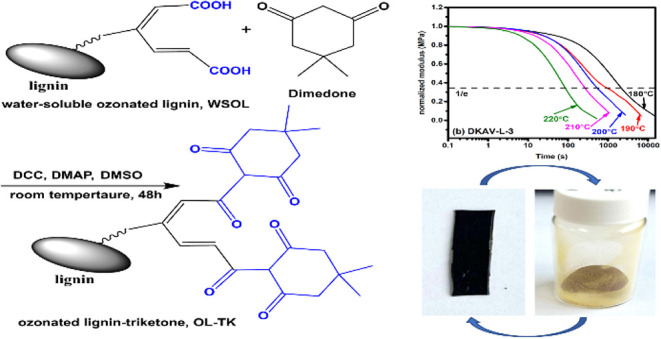

## Introduction

Vitrimers possess associative covalent adaptable networks (CANs), which could exhibit topology changes through bond-exchanging reactions.^1–3^ The unique features make vitrimers exhibit some attractive properties, such as recycling, reprocessing, and self-healing behaviors, which could not be conducted with conventional cross-linked polymers. These properties of vitrimers make the thermosetting resins become thermoplastics-like and provide possible approaches to address the concerns of the circular economy and zero CO_2_ emission.^4^ Moreover, to further increase the “green” extent of vitrimers, employing biomass-based and recycled raw materials in the preparation of vitrimers is noteworthy and has received much research attention.^5,6^ Lignin is a major naturally abandoned material obtained from plants.^7^ It has been widely used as a precursor in the production of biomass-based chemicals such as vanillin, syringaldehyde, eugenol, *p*-coumaric acid, and many alkylphenols, which have been utilized as raw materials for the production of low-carbon materials.^7^ Moreover, as lignin possesses chemically active aromatic and aliphatic −OH groups, lignin itself could be a suitable raw material for the preparation of the corresponding polymeric materials. Consequently, lignin-based vitrimers have been studied for the development of low-carbon polymers with attractive dynamic properties.^8–10^

Zhang and coworkers^11^ utilized ozonated lignin, which possessed some −COOH groups, as a cross-linking agent for an ester-containing epoxide compound to result in epoxy vitrimers containing dynamic ester bonds. Moreno et al.^12^ prepared vitrimers of lignin and poly­(ethylene glycol) divinyl ether through the PhOH/vinyl click reaction. The products possessed dynamic acetal bonds and were used as recoverable adhesives. Si and coworkers^13^ utilized tri­(ethylene glycol) divinyl ether to be polymerized with lignin and studied the photothermal conversion properties of the prepared lignin vitrimer-based adhesives. Dynamic borate ester bonds were also employed in the lignin-based vitrimers, which demonstrated intrinsic photoconversion property and high photothermal remoldability for adhesive applications.^14^ Avérous and coworkers^15^ recently reported the lignin-based vitrimers possessing dynamic vinylogous urethane linkages, which demonstrated a closed-loop recycling feature. Jin et al.^16^ reported lignin-based vitrimers possessing dual dynamic bonds (hydroxyl-ester and β-amino ester) to show photothermal characteristics and healing ability. The above-mentioned examples were mostly conducted with direct polymerization of lignin by other reagents and chemical modification of lignin to introduce various dynamic bonds into the CANs of the lignin-based vitrimers. In this work, the dynamic diketoenamine linkages are applied to prepare lignin-based vitrimers^17–22^ for the first time. The formation of diketoenamine linkages is conducted through the reaction between a triketone and a primary amine.^17^ The dynamic feature of diketoenamine linkages is performed through the exchange reaction between diketoenamine and primary groups.^17–22^ Hence, the formation of associative CANs containing dynamic diketoenamine linkages is conducted through cross-linking reactions between multifunctional triketone and primary compounds with loading excess primary amine groups. Compared to other lignin-containing vitrimers, the diketoenamine-based lignin vitrimers might exhibit some attractive features such as polymerization at mild conditions, mild conditions for exchange reactions, and significant acid-catalyzed hydrolysis for chemical recycling.^17^

Synthesis of a multifunctional triketone compound from a precursor possessing multifunctional carboxylic acid groups could be the first step of the chemical synthesis routes for diketoenamine vitrimers.^17,22^ In this work, water-soluble lignin (WSOL)^23^ possessing some carboxylic acid groups obtained from the ozonation of lignin and suitable separation was used as the starting material. Triketone groups were then incorporated into the ozonated lignin. The obtained product (OL-TK) was then reacted with a trifunctional primary amine compound (tris­(2-aminoethyl)­amine, TREN)^17^ to result in the corresponding lignin-based vitrimer possessing dynamic diketoenamine linkages. Moreover, OL-TK has also been utilized as a reactive modifier for conventional diketoenamine-based vitrimers. The relatively rigid lignin structure brought significant effect on restricting the chain mobility so as to depress the creep behavior of the vitrimers,^24^ especially at the temperatures above the topology freezing transition temperature (*T*_*v*_) and glass transition temperature (*T*_*g*_) of the vitrimers, where the polymer networks gained sufficient chain mobility.

## Results and Discussion

### Synthesis of Triketone Derivative of Lignin

Diketoenamine linkages could be generated by the reaction between the triketone and amine groups. Hence, the triketone derivative of lignin was first synthesized and characterized for the introduction of lignin to diketoenamine-based polymers. As shown in Scheme 1, the first step was ozone treatment on lignin to introduce some carboxylic acid groups to lignin molecules. The water-soluble fraction of the ozonated lignin (WSOL) was separated and collected.^23^ The carboxylic acid content of WSOL was measured with titration to be 2.73 mmol g^–1^. Incorporation of triketone groups onto lignin was conducted through the reaction between WSOL and dimedone. The obtained product was coded as OL-TK and subjected to characterization (Figure S1). In FTIR measurements, WSOL exhibited the absorption of carboxylic acid groups at 2570 and 1740 cm^–1^. The absorption still appeared in the OL-TK FTIR spectrum with relatively weak intensities. OL-TK showed an additional absorption at about 1650 cm^–1^ assigned to be the C=O of triketone groups.^17^ Together with the absorptions of methyl groups at 2940 and 2870 cm^–1^, the results provided preliminary support for the presence of triketone groups in the OL-TK product. The resonance peaks at δ = 0.99 and δ = 2.24 ppm appearing in the ^1^H NMR spectrum of OL-TK were assigned to be associated with the methyl and methylene groups of triketone, respectively. OL-TK showed the signal of −COO ***H*** at about δ = 12.7 ppm, which exhibited a peak shift and a reduced intensity compared to the signal of −COO ***H*** found with WSOL. Christensen and coworkers^17^ reported the characteristic resonance peak of triketone groups (the tertiary C– ***H*** linked to 3 ketone groups) at about δ = 18.11 ppm. Although a weak signal at δ = 17.4 ppm appeared in ^1^H NMR of OL-TK, the relatively low intensity of the signal might not give a satisfactory support to the presence of the triketone groups. The results might be attributed to the low conversion of the triketone-formation reaction and the possible poor solubility of OL-TK in the solvent. The total amount of triketone and −COOH groups of OL-TK determined with titration was 3.38 mmol g^–1^, which was higher than the value measured with WSOL. During the preparation process, the samples having relatively low −COOH contents owned relatively poor solubility in water so they were not collected. As the triketone/–COOH molar ratio of OL-TK read from ^1^H NMR analysis was about 0.44, OL-TK owned a triketone content of 1.03 mmol g^–1^. The discussion presented above supports the successful synthesis of triketone-functionalized lignin (OL-TK). The presence of the triketone groups makes OL-TK a suitable reactant with TREN for the preparation of a lignin-based diketoenamine resin.

**Scheme 1:**
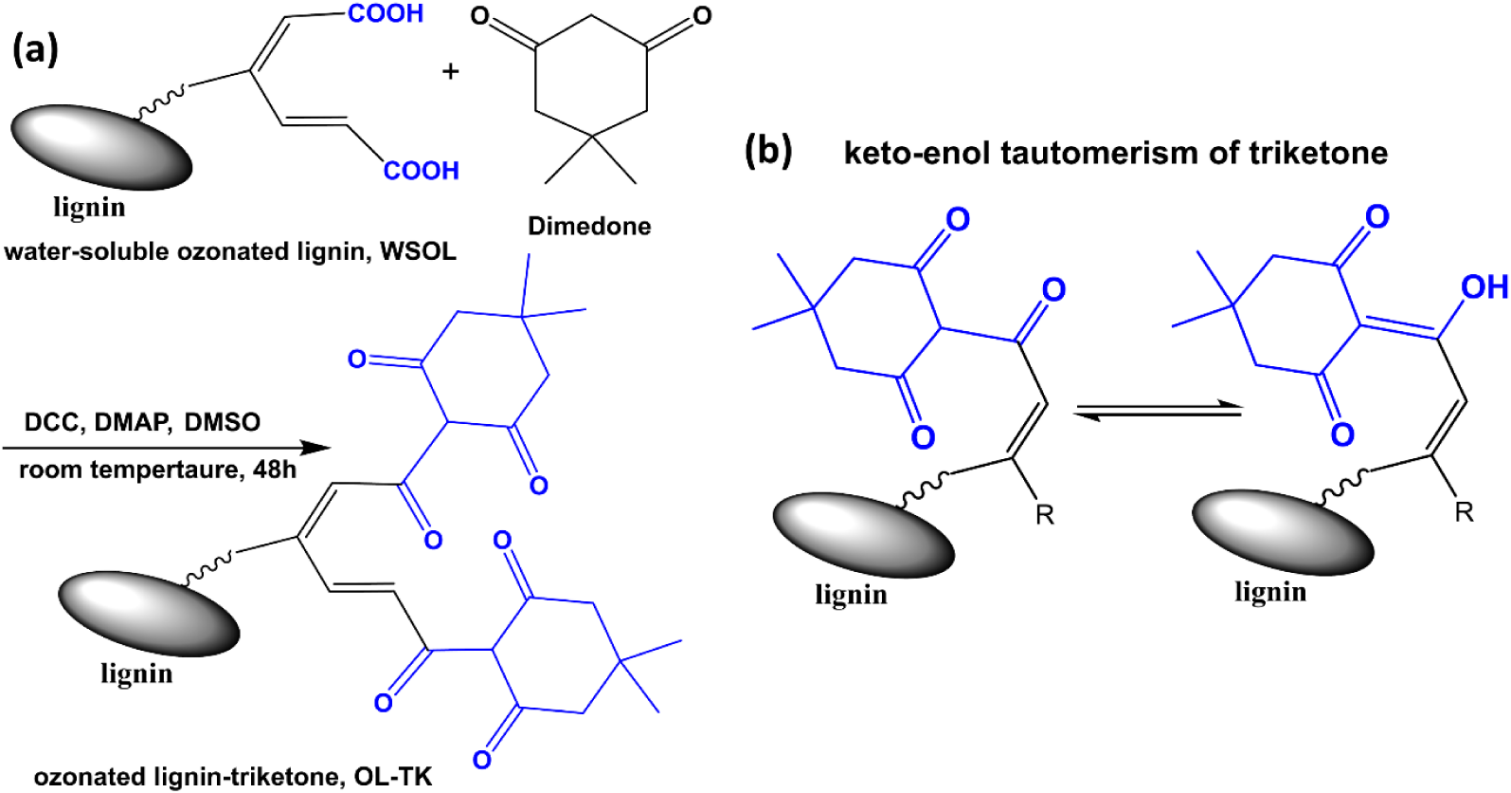
(a) Synthesis of Triketone-Modified Lignin (OL-TK) and (b) Keto–Enol Tautomerization of Triketone Groups

### Cross-Linked Products of OL-TK and TREN

Taking advantage of the reaction between triketone and primary amine,^17^ cross-linked products of OL-TK and TREN were prepared (Scheme 2) through the thermal process mentioned in the Experimental Section. The lignin-based cross-linked resin with 2 times the excess primary amine was obtained and coded as OLTK-CR-2. OLTK-CR-2 showed a gel fraction of 95 wt % measured in dimethyl sulfoxide (DMSO) at 25 °C to demonstrate its highly cross-linked structure. The comparative sample prepared with WSOL and TREN (WSOL-CR-2) showed a gel fraction of 58 wt % in the test. The results indicate that the triketone/amine addition reaction (OLTK-CR-2), compared to the −COOH/amine amidation reaction for WSOL-CR-2, is effective for cross-linking the employed reactants in the sample preparation process. Nevertheless, the results still suggest that both triketone/amine and −COOH/amine reactions might be involved in the cross-linking reactions between OL-TK and TREN. OLTK-CR samples prepared with various excess amine groups were also prepared. Nevertheless, the gel fractions found with OLTK-CR-1.5 and OLTK-CR-2.5 were 91 and 92 wt %, respectively. Hence, OLTK-CR-2, which exhibited the highest gel fraction among the samples, was applied for further examination.

**Scheme 2:**
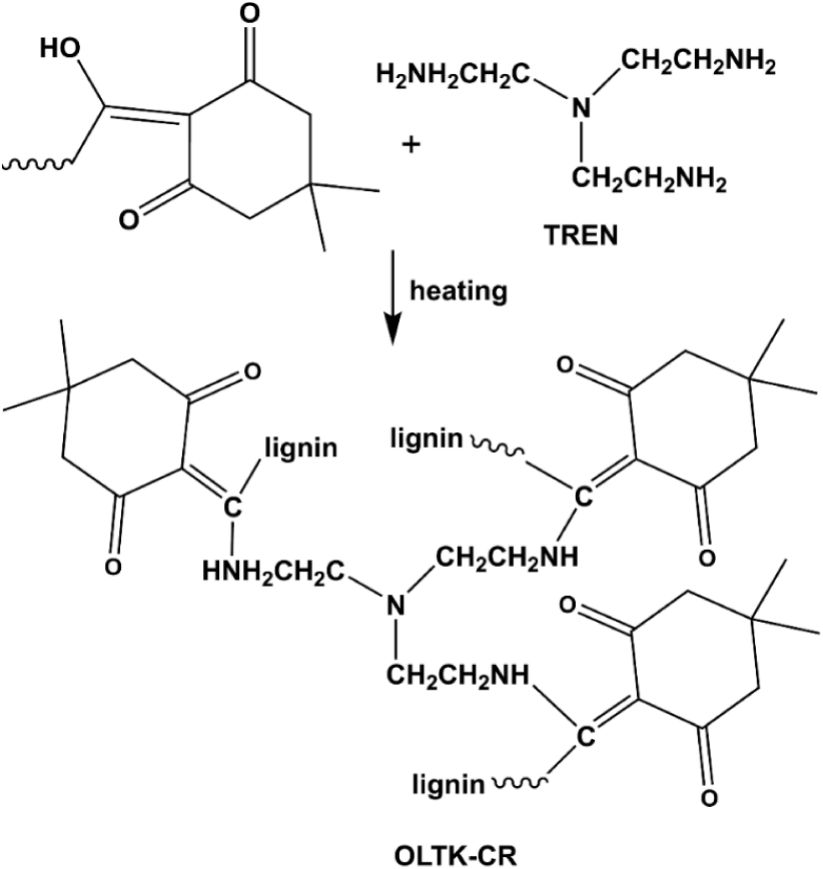
Cross-Linking Reaction between OL-TK and TREN through the Triketone-Amine Addition Reaction to Result in OLTK-CR Resin

As the FTIR spectra shown in Figure S2, the diketoenamine groups exhibited absorption peaks at 1643 and 1567 cm^–1^. Moreover, based on the prior studies conversion of triketone to diketoenamine groups would result in a blue shift and red shift to C=C and C=O absorption, respectively.^17^ The results were still observed with OLTK-CR-2. Compared with the absorption of C=O groups at 1650 cm^–1^ for OL-TK, OLTK-CR-2 exhibited the absorption of C=O groups at 1643 cm^–1^. Nevertheless, due to the overlapped absorptions, the blue shift for the C=C absorptions between OL-TK and OLTK-CR-2 could not be clearly assigned. Thermal characterization results of OLTK-CR-2 are collected in Figure 1. OLTK-CR-2 exhibited a glass transition temperature (*T*_*g*_) of about 165 °C in differential scanning calorimetric (DSC) measurement, which is somewhat higher than the *T*_*g*_ (128 °C) reported to the 2,5-furandicarboxylic acid (FDCA)-based diketoenamine resin^22^ due to the relatively rigid structure of lignin. Moreover, the *T*_*g*_ of OLTK-CR-2 is much higher than the values reported for other lignin-based vitrimers, such as lignin-epoxy vitrimers (133 °C),^11^ lignin vitrimers possessing borate ester bonds (76 °C),^14^ and lignin-based vitrimers containing both β-amino ester and hydroxyl ester bonds (47 °C).^16^ The rigidity of cyclic diketoenamine linkages contribute to increase the glass transition temperatures of the lignin-diketoenamine vitrimers. In thermogravimetric analysis (TGA), the sample gave initial thermal weight loss at about 200 °C. The retarded weight loss behavior after 300 °C indicated the formation of thermally stable residuals after initial thermal degradation, which is attributed to the aromatic structure of lignin. The aromatic structure of lignin still contributed to about 10 wt % residual at 800 °C, since the previously reported FDCA-based analog showed a full degradation at 800 °C.^22^ Regrettably, the obtained OLTK-CR-2 cracked in the cooling process due to its high brittleness, which was attributed to the high rigidity and high weight fraction (79 wt %) of OL-TK in the prepared sample. Moreover, the lack of long and flexible chains in the TREN reagent still contributes to the high brittleness of OLTK-CR-2.^11–16^ Consequently, measurements of the mechanical properties of OLTK-CR-2 were not successfully conducted due to the poor preparation of a suitable specimen. OLTK-CR-2 was then ground into a powder and thermally recycled at 180 °C and 14 MPa for 2 h. The obtained sample (OLTK-CR2/R1) still cracked under cooling and possessed some defects due to its high brittleness (Figure S3). It is regrettable that the thermal recycling of OLTK-CR-2 was not as successful as for other vitrimers due to its high lignin content. A possible modification on OLTK-CR-2 to reduce its brittleness could be employing other primary amine cross-linking agents possessing flexible segments, such as poly­(tetrahydrofuran)-bis-tris-2­(aminoethyl)­amine reported by Dailing et al.^25^ On the other hand, OLTK-CR-2 was completely dissolved in 1.0 N HCl solutions of DMSO and THF/water (50/50 in w/w). As OLTK-CR-2 showed a high gel fraction in DMSO, the dissolution behavior was attributed to the acid-catalyzed degradation of the diketoenamine bonds^17^ and indicated the feasibility of chemical recycling of OLTK-CR-2 (Figure S3). The acid-catalyzed hydrolysis reaction and enamine–enol tautomerism of diketoenamine are shown in Figure S4.

**Figure 1:**
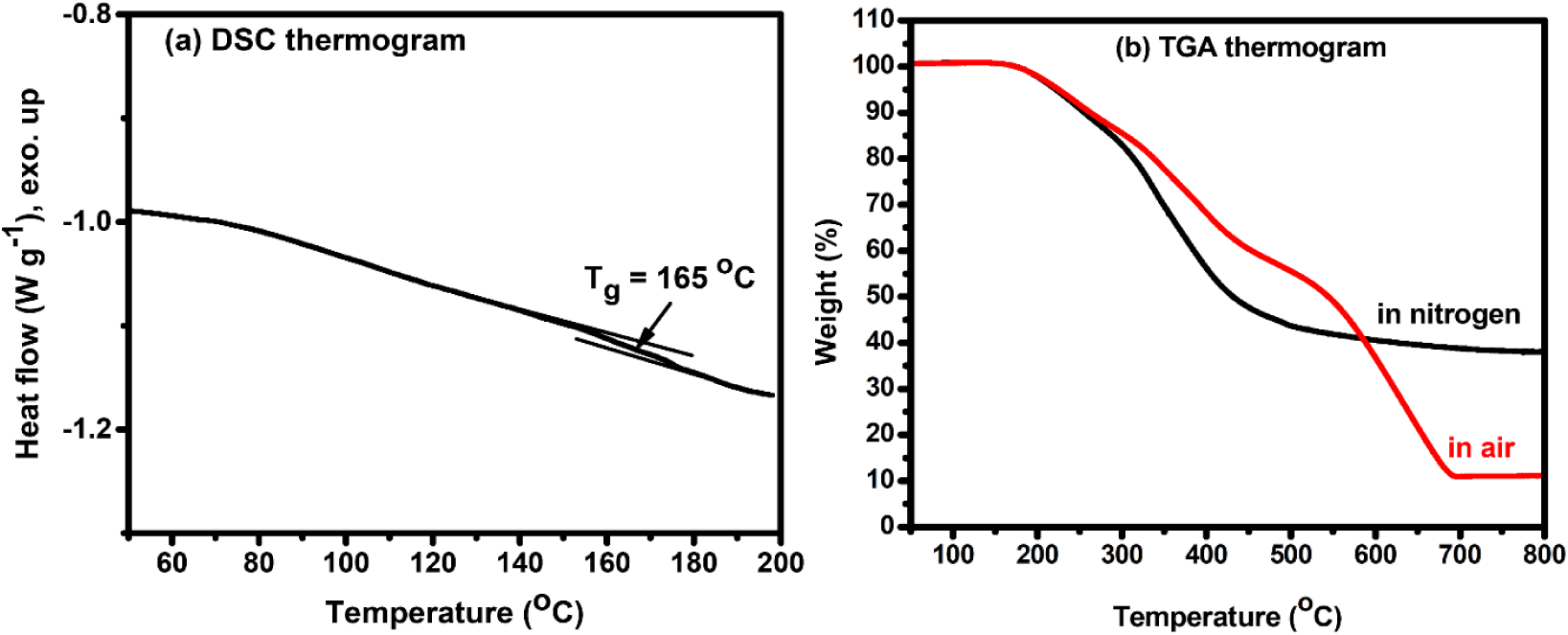
(a) DSC and (b) TGA thermograms recorded on the OLTK-CR-2 resin.

### OL-TK as a Reactive Modifier for Diketoenamine-Based Vitrimers

In addition to being utilized as a monomer in the preparation of lignin-based polymers, lignin could also be used as a reactive additive for conventional resins.^26,27^ OL-TK was then utilized as a reactive modifier for the diketoenamine-based vitrimer prepared with a furan-triketone compound (FTK) and TREN (Figure S5).^22^ In the previous work, the prepared TREN/FTK resin showed further polymerization behavior in the thermal recycling process, indicating the relatively low reaction conversion with the original cross-linking process.^22^ Hence, in this work an additional thermal treatment step was applied to the samples obtained from the polymerization between TREN and FTK. Consequently, the prepared TREN/FTK sample could be relatively similar to the TREN/FTK-R1 sample (thermally recycled sample from TREN/FTK) reported in the prior work.^22^

The prepared samples (OL-TK modified TREN/FTK diketonenamine vitrimers) were coded as DKAV-L-*X*, where *X* (*X* = 0 to 10 in this study) denotes the added amount of OL-TK in wt %. The excess of amine compared to triketone groups in the compositions was 7 mol %. DKAV-L-0 (the pristine TREN/FTK resin) gave a gel fraction of 98.1 wt % measured with DMSO at 30 °C. The addition of OL-TK did not alter the cross-linking reactions since all the OL-TK modified samples possessed high gel fractions above 95 wt %. The relatively low gel fraction of 95.4 wt % found with DKAV-L-10 might be attributed to the steric hindrance of the bulky lignin units of OL-TK. The degrees of swelling of the samples were also obtained with a similar test manner. Compared to the values measured with DKAV-L-0 (1.2 wt %) and DKAV-L-3 (0.9 wt %), the relatively high degrees of swelling found with DKAV-L-7 (14.2 wt %) and DKAV-L-10 (14.8 wt %) suggested the 2 samples possess relatively loose cross-linked structures contributed with the bulky lignin units, since all samples have similar chemical structures. In SEM micrographs (Figure S6), all samples exhibited dense and homogeneous surfaces at low magnification in cross-sectional view. Nevertheless, the added OL-TK exhibited submicrometer domains in the DKAV-L-*X* samples. The domains became relatively obvious with increasing OL-TK contents. The lignin domains brought a plasticizing effect to the vitrimers to result in decreases in their *T*_*g*_ measured with DSC (Figure 2). Compared to the *T*_*g*_ of 145 °C of DKAV-L-0, the OL-TK modified vitrimers showed relatively low *T*_*g*_’s of 136–138 °C. Nevertheless, the OL-TK modification did not alter the thermal stability of the vitrimers, as all samples exhibited similar temperatures at 5 wt % weight loss (236–239 °C) and char residuals at 800 °C (36.7–38.8 wt %) measured with a thermogravimetric analyzer (TGA) in nitrogen. In dynamic mechanical analysis (DMA), all the vitrimers exhibited a similar storage modulus of about 2–3 GPa at room temperatures.

**Figure 2:**
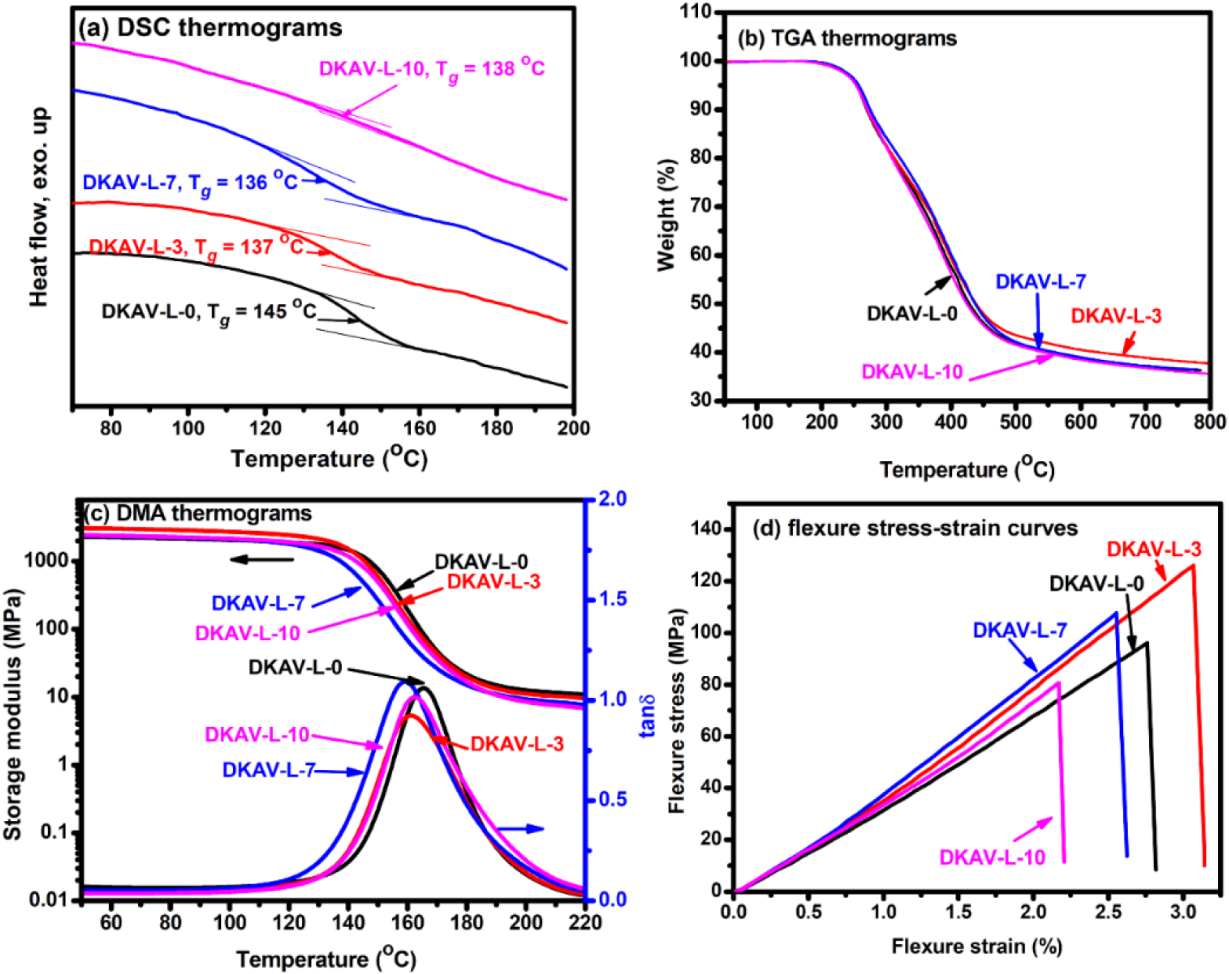
Thermal and mechanical measurements on the DKAV-L-*X* samples: (a) DSC thermograms, (b) TGA thermograms recorded in a nitrogen atmosphere, (c) DMA thermograms, and (d) flexural stress–strain curves.

The *T*_*g*_’s values read from the temperatures of tanδ peaks are somewhat higher than the values measured with DSC, and the relative orders are similar to those from DSC measurements (Table 1). Moreover, the OL-TK modification resulted in decreases in the storage modulus at the rubber plateau regions of the vitrimers (Table 1), indicating that the modified vitrimers owned relatively low cross-linking densities compared to the pristine vitrimer. The cross-linking densities of DKAV-L-0, DKAV-L-3, DKAV-L-7, and DKAV-L-10 were 0.93, 0.84, 0.72, and 0.61 mmol cm^–3^, respectively. Hence, OL-TK modification resulted in both a plasticizing effect and multiple functionality to the vitrimers. The 2 factors brought trade-off actions on the chain mobility of the vitrimers to demonstrate the *T*_*g*_’s and cross-linking densities of DKAV-L-7 and DKAV-L-10 as discussed above.

**Table 1:** Mechanical Properties of the DKAV-L-*X* Samples

	DMA measurements			Flexural tests		
Sample	*T*_*g*_ (^o^C)	Storage modulus at rubbery plateau (MPa)	Cross-linking density (mmol cm^–3^)	Flexural modulus (MPa)	Flexural stress at break (MPa)	Flexural strain at break (%)
DKAV-L-0	165	11.6	0.93	4189 ±185	99.6 ±8.2	2.6 ±0.26
DKAV-L-3	162	10.3	0.84	4448 ±318	129.3 ±7.3	3.3 ±0.39
DKAV-L-7	160	8.7	0.72	4965±295	110.5±9.8	2.6±0.23
DKAV-L-10	163	7.4	0.61	4123±326	83.9±5.6	2.5±0.37

The effect of the OL-TK modification on the mechanical properties of the diketoenamine-based vitrimers was probed with flexural tests. A reinforcement effect was observed with the addition of OL-TK, as both DKAV-L-3 and DKAV-L-7 showed relatively higher flexural stress and flexural modulus than did DKAV-L-0. Compared to DKAV-L-0, DKAV-L-3 exhibited 30% and 27% increases in the flexural stress and flexural strain, respectively. The results indicated that a suitable amount of OL-TK might simultaneously reinforce and toughen the diketoenamine vitrimers. Nevertheless, the high content of OL-TK in DKAV-L-10 did not increase its mechanical properties due to lignin aggregation and the induced plasticizing effect.

### Dynamic Behaviors and Physical Recycling of the Diketoenamine Vitrimers

The effect of OL-TK modification on the dynamic behaviors of the vitrimers has been examined. Figure 3 shows the stress relaxation behavior of the vitrimers measured at a strain of 0.5% determined from strain sweep measurements (Figure S7). The original stress relaxation curves are also included in Figure S8. All the samples exhibited significant stress relaxation behaviors at 180–220 °C being attributed to the performance of the diketoenamine/amine exchange reaction. The addition of 3 wt % OL-TK brought an effect on the restriction of the chain mobility so as to result in a prolonged relaxation time from 2660 to 3090 s at 180 °C. This effect became minor at high temperatures due to the high chain mobility at high temperatures. The vitrimers possessing high OL-TK contents (DKAV-L-7 and DKAV-L-10) exhibited relatively short relaxation time compared to the pristine vitrimers. This effect could be attributed to the acid-catalytic effect on the diketoenamine/amine exchange reaction contributed by the −COOH groups of OL-TK.^28^ The catalytic effect was still supported with the relatively low activation energy of stress relaxation measured with DKAV-L-10 (142 kJ mol^–1^) compared to the value of 159 kJ mol^–1^ of DKAV-L-0. Compared to the vitrimers based on aliphatic triketone compounds and TREN, the DKAV-L-*X* vitrimers still exhibit relatively long stress relaxation time and high temperatures required for stress relaxation due to the low chain mobility^17,19^ As *T_v_* indicates the lowest temperature at which the CANs‘ topology changes through bond-exchanging reactions, a low *T_v_* indicates the high dynamic features at low temperatures. However, a low *T_v_* might also suggest a low service temperature of the vitrimers. The *T*_*v*_’s of the vitrimers were determined with the temperatures possessing a viscosity of 10^12^ Pa s.^7,29^ DKAV-L-0 showed a *T_v_* of 146 °C, which was close to the value of 149 °C found with DKAV-L-3 and much higher than the values of DKAV-L-7 (136 °C) and DKAV-L-10 (139 °C). The measured *T_v_*’s of the vitrimers were in the glass transition regions of the samples. Hence, both polymer chain mobility and diketoenamine/amine exchange reaction rate might contribute to the stress relaxation behaviors.^30^ At temperatures below *T*_*g*_ and *T*_*v*_, rigid vitrimers usually exhibit low initial strains and creeps due to restricted chain mobility and bond-exchanging behaviors. Hence, all of the OL-TK-modified diketoenamine vitrimers showed very low initial strains and creeps at 80 °C (Figure 4). Nevertheless, the initial strains and creeps increased with increasing the testing temperatures. At 120 °C, DKAV-L-0 showed a creep of about 1.9% and a strain recovery of 37%. The creep was depressed to 0.7% with a strain recovery of 34% with addition of 3 wt % of OL-TK. Moreover, relatively high OL-TK contents promote the bond-exchanging reactions as the discussion on stress relaxation behaviors. High creeps were observed with both DKAV-L-7 and DKAV-L-10. The creep-depressing effect was still observed with DKAV-L-3 at 140 °C. However, the extent of creep-depressing was not as significant due to the high bond-exchanging rates and less restriction on chain motion. The above results indicate that OL-TK modification enhanced the dynamic features of the vitrimers for the cases of high OL-TK contents. For the sample possessing 3 wt % of OL-TK, the effect of OL-TK on promoting the bond-exchanging reactions was not as significant. On the other hand, the presence of OL-TK exhibited some effect on depressing the creep of vitrimers.

**Figure 3:**
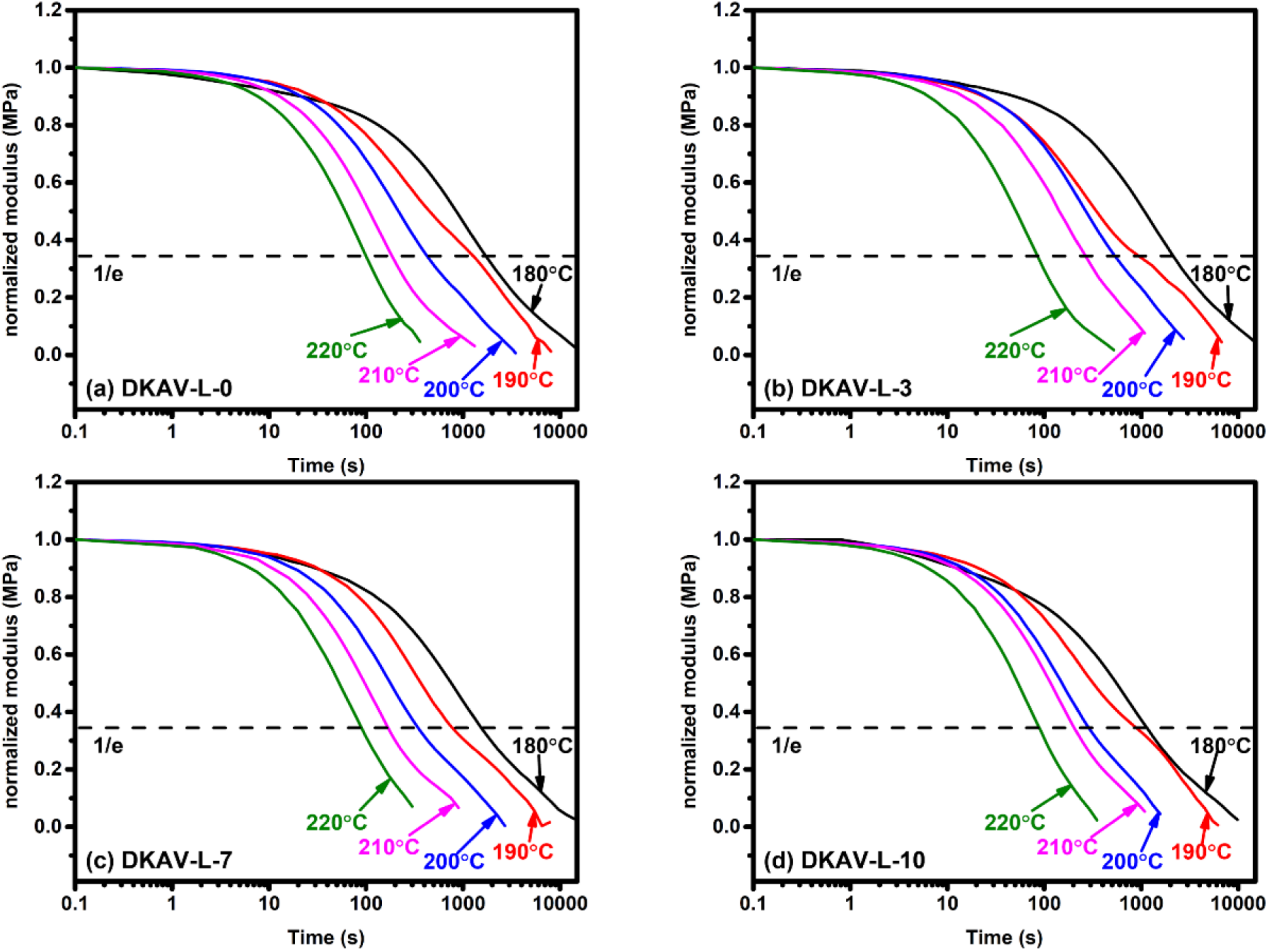
Normalized stress relaxation curves recorded on (a) DKAV-L-0, (b) DKAV-L-3, (c) DKAV-L-7, and (d) DKAV-L-10 samples at different temperatures.

**Figure 4:**
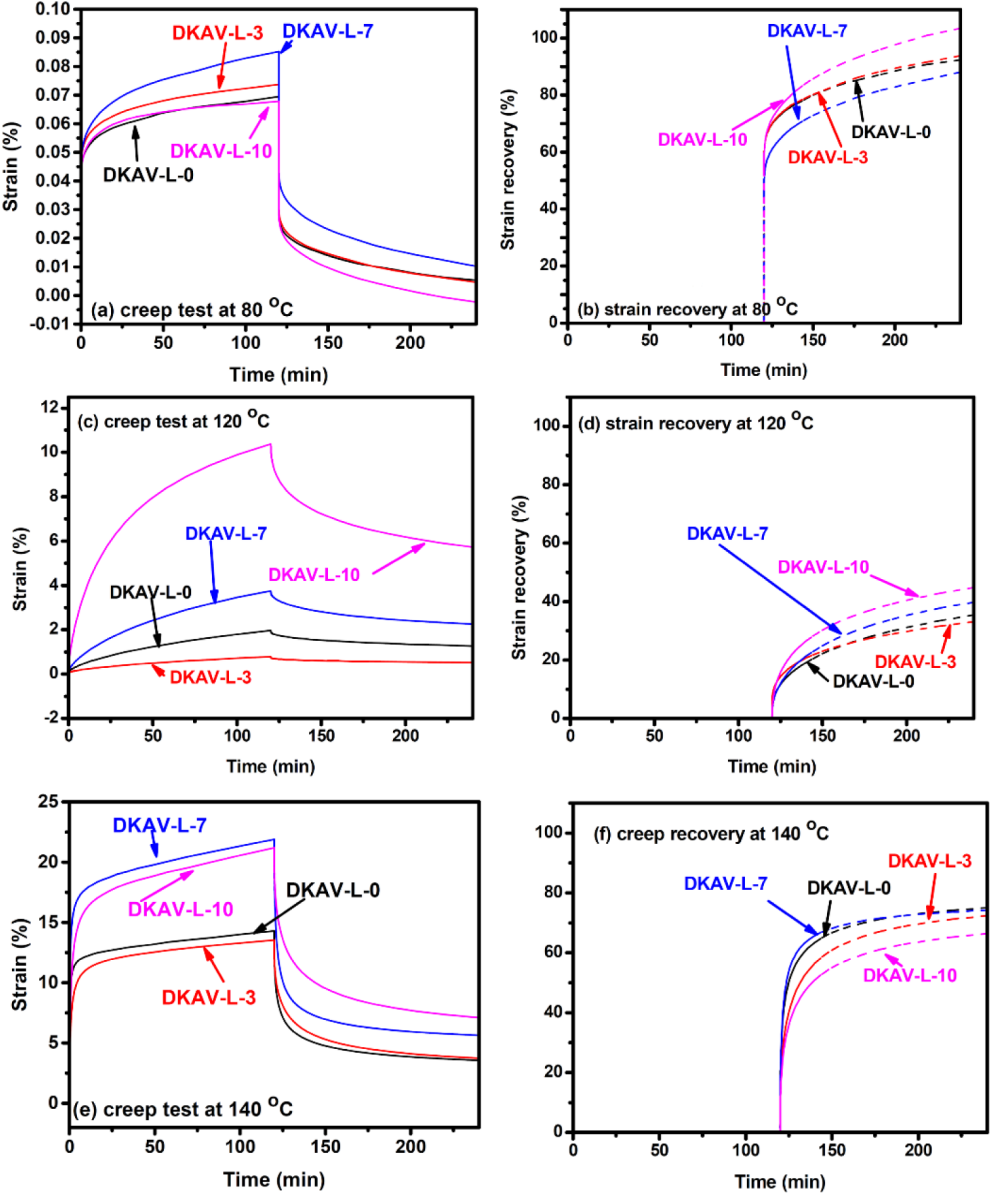
Creep tests and strain recovery recorded on DKAV-L-3, DKAV-L-7, and DKAV-L-10 at (a, b) 80 °C, (c, d) 120 °C, and (e, f) 140 °C.

Based on the above results and discussion, DKAV-L-3 and DKAV-L-7 were taken as the samples for further tests on physical recycling and thermal reprocessing. The data recorded on DKAV-L-0 were also obtained for a comparison. The original samples were ground into powders and then thermally reprocessed at 190 °C and 14 MPa for 20 min (Figure S9). As the results observed with vitrimers, the 3 samples showed good behaviors in thermal recycling and reprocessing. In SEM observation (Figure 5), both DKAV-L-0/R1 and DKAV-L-3/R1 did not exhibit obvious cracks and defects in the recorded micrographs, indicating that the powders fused and merged together in a good manner. Nevertheless, the relatively high content of OL-TK in DKAV-L-7 resulted in poor interfacial compatibility and cracks observed with the cross-sectional view of the DKAV-L-7/R1 sample, although DKAV-L-7 still exhibited a high extent of stress relaxation at 190 °C.

**Figure 5:**
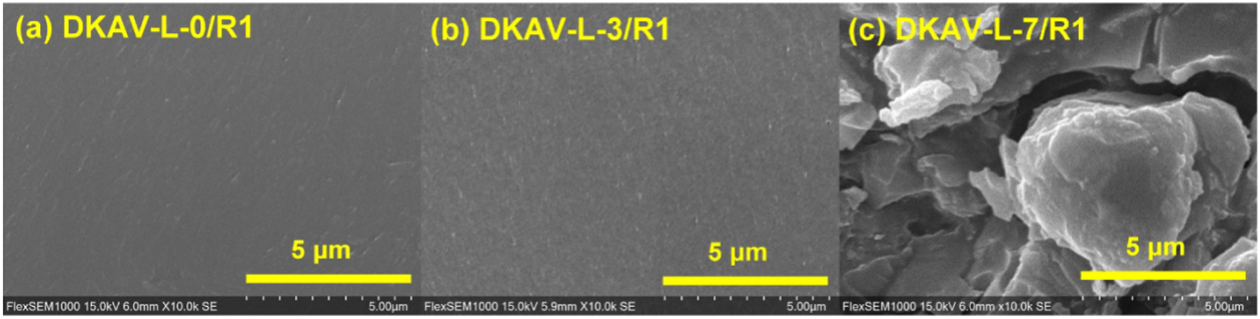
SEM micrographs recorded on the recycled samples of (a) DKAV-L-0, (b) DKAV-L-3, and (c) DKAV-L-7.

In TGA measurements (Figure 6), the thermograms recorded on the recycled samples almost overlapped with the thermograms of the individual original samples. The gel fractions of DKAV-L-0/R1 and DKAV-L-3/R1 measured in DMSO were 97.7% and 98.1%, respectively, suggesting that the recycled samples still possess high cross-linking densities.

**Figure 6:**
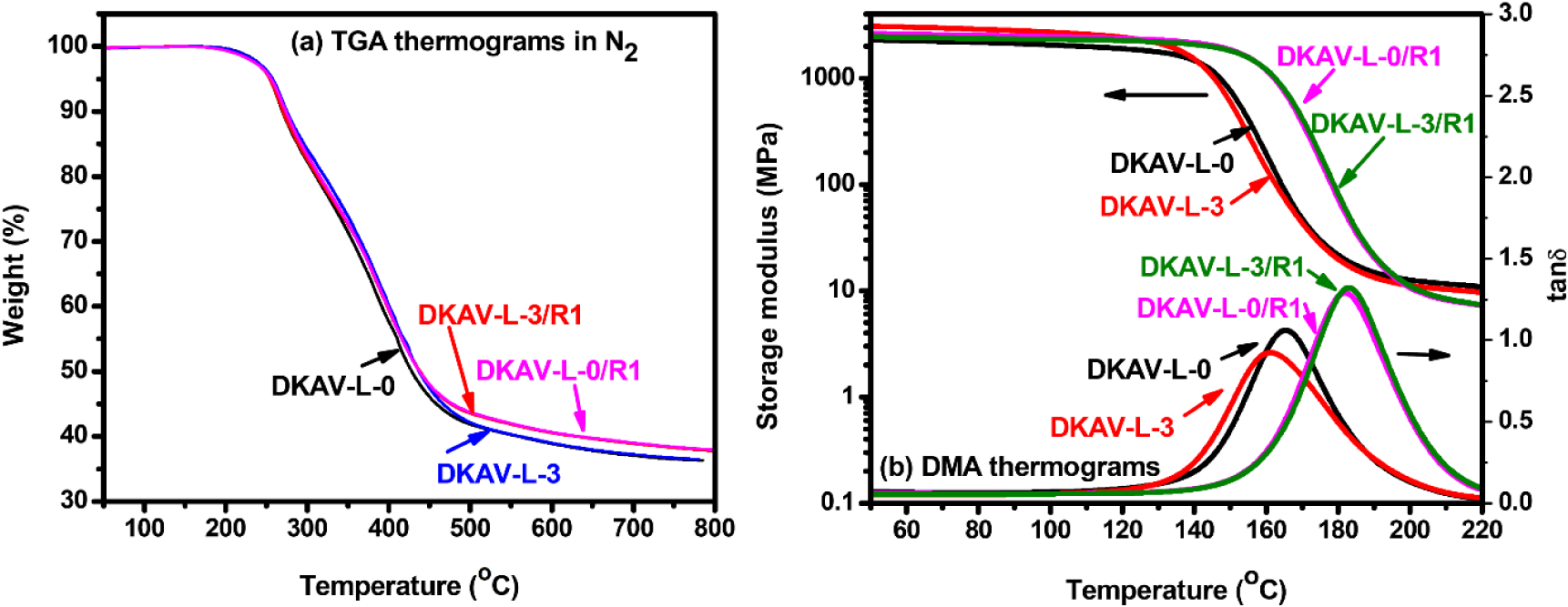
(a) TGA (measured in a N_2_ atmosphere) and (b) DMA thermograms recorded on recycled samples of DKAV-L-0/R1 and DKAV-L-3/R1. The thermograms recorded on the original samples (DKAV-L-0 and DKAV-L-3) being included for comparison.

Nevertheless, the *T*_*g*_’s of the vitrimers increased significantly after the recycling processes. DKAV-L-0/R1 and DKAV-L-3/R1 gave *T*_*g*_’s of 182 and 183 °C, respectively, which were about 30 °C higher than the *T*_*g*_’s recorded on the original samples (Figure 6b). The results indicated that further cross-linking reactions might take place in the thermal recycling processes. Nevertheless, for the recycled samples, the presence of 3 wt % OL-TK did not alter the *T*_*g*_ and dynamic mechanical properties of the vitrimers. The relatively high *T*_*g*_ and cross-linking density of the recycled samples (DKAV-L-0/R1 and DKAV-L-3/R1) suggest the high restriction of chain mobility limiting the efficiency in further recycling.

Moreover, although the relatively high OL-TK content in DKAV-L-7 enhanced the chain mobility and stress relaxation, as discussed above, the thermal recycling tests conducted on DKAV-L-7 did not give positive results. DKAV-L-7/R1 showed some heterogeneous regions with aggregated lignin domains and defects in the SEM micrographs (Figure 5), suggesting the relatively poor thermal recycling efficiency of DKAV-L-7 although DKAV-L-7/R1 still exhibited sufficient stress relaxation at 190 to 210 °C with a relatively short stress relaxation time compared to the original DKAV-L-7. The reaction-induced phase separation between the diketoenamine resin and lignin might be attributed to the fact that OL-TK did not possess amine groups to be involved in the bond-exchanging reactions. The bond-exchanging reaction might reduce the density of chemical linkages between diketoenamine and lignin, resulting in the phase separation. One possible approach to address this issue and enhance the interfacial compatibility between OL-TK and the matrix resin might be the incorporation of amine groups to OL-TK to make OL-TK involve in the bond-exchanging reactions. In addition, the *T*_*g*_ measured with DKAV-L-7/R1 was 178 °C, which was lower than those of DKAV-L-0/R1 and DKAV-L-3/R1. Hence, the OL-TK in DKAV-L-7 and DKAV-L-7/R1 acted as plasticizers rather than reinforcing agents due to the phase separation effect mentioned above.

## Conclusions

The multiple carboxylic acid groups of ozonated lignin make it a suitable biomass-based raw material for the preparation of the lignin-triketone derivative and the corresponding diketoenamine vitrimers. Unlike the utilization of epoxidized lignin in the preparation of epoxy resin-based vitrimers,^11^ the lignin-based diketoenamine resins showed relatively high rigidity and brittleness due to the resins did not possess flexible chemical segments and due to their high cross-linking densities contributed by the multifunctional lignin precursors. On the other hand, the synthesized lignin-triketone compound was employed as a reactive modifier for furan-based diketoenamine resins.^22^ The addition of 3 wt % of OL-TK might partially depress the creep of the vitrimers at high temperatures and retained the thermal recycling feature of the vitrimers. Nevertheless, the addition of high fractions of OL-TK (7 wt %), although retaining the stress relaxation behaviors of the modified vitrimers, exhibited phase separation in the vitrimers to result in relatively poor thermal recycling property and to bring a plasticizing effect to the vitrimers. The phase separation might be attributed to the poor interfacial compatibility between OL-TK and the resin matrix as the OL-TK did not possess amine groups to perform bond-exchanging reactions with the diketoenamine resins. Further studies might involve the incorporation of TREN moieties into the lignin rather than the functionalization of lignin with triketone groups conducted in this work.

## Experimental Section

### Materials

Lignin was purchased from Tokyo Chemical Industry Co., Ltd. (TCI, product number: L0045) and used as received. The product was a dealkalized lignin obtained from sodium ligninsulfonate after partial desulfonation, oxidation, hydrolysis, and demethylation. Preparation of water-soluble lignin (WsOL) was conducted in the laboratory according to the reported method.^23^ FTK was prepared from furanic dicarboxylic acid (Combi-Blocks Inc.) and 5,5-dimethylcyclohexane-1,3-dione (TCI, dimedone, >99%) following the method reported in our previous work.^22^ 4-Aminodimethylpyridine (DMAP) and *N*,*N*′-dicyclohexylcarbodiimide (DCC) were received from Thermo Fisher Co. TREN was a commercial product from Acros and was dried over molecular sieves prior to use.

### Instrumental Methods

The PerkinElmer Spectrum II FTIR spectrometer was employed in recording FTIR spectra of the samples. The measurements were conducted with a wavenumber range of 4000–400 cm^–1^.

Nuclear magnetic resonance (NMR) measurements were conducted with a Brüker Avance NMR spectrometer (500 MHz) with tetramethylsilane as an internal reference. To prevent water interference in quantitative measurements, the sample was dried at 70 °C under a vacuum overnight. Dry deuterated dimethyl sulfoxide (DMSO-D_6_, a commercial product from Sigma-Aldrich) was employed as a solvent for the preparation of the sample solutions. Thermal analysis instruments from Thermal Analysis Co. were employed in the measurements, including differential scanning calorimetry (TA-Q20 DSC, in nitrogen, heating rate: 10 °C min^–1^), thermogravimetric analysis (TA-Q50 TGA, in nitrogen and air, heating rate: 10 °C min^–1^, and dynamic mechanical analysis (TA-Q800 DMA, tensile mode, heating rate: 3 °C min^–1^). DSC measurements were performed with a heating rate of 10 °C min^–1^ from 50 to 200 °C. The measured *T*_*g*_ read from the DSC curve was determined with an inflection method. The general DMA measurements were performed with a frequency sweep from 0.01 to 100 Hz, a constant force of 0.01 N, and an amplitude of 0.1%. Strain sweep tests from 0.01 to 1.4% were measured at a frequency of 1 Hz and a constant force of 0.01 N at 180 °C. Cross-linking densities of the measured samples were calculated from the equation of *V*_*c*_ = *E*′/3*RT*, where *R* is the gas constant, *T* is the temperature of 50 K above the *T*_*g*_ of the sample, *E*′ is the storage modulus at *T* temperature.^31^ Stress relaxation measurements were performed at different temperatures with a fixed strain of 0.5%. With an applied stress of 1.0 MPa, the creep tests were carried out at various temperatures. Flexural stress–strain tests were performed with an Instron 5543 Analyzer at a bending rate of 2.0 mm min^–1^. The sample size was 25 × 5 × 0.5 mm^3^. SEM pictures were obtained with a Hitachi S-4800 field-emission SEM.

### Synthesis of OL-TK

In 50 mL of DMSO, dimedone (15.0 g, 104 mmol), WsOL (1.5 g, 4.1 mmol), and DMAP (6.1 g, 50 mmol) were dissolved to form a homogeneous solution. Excess dimedone was employed to increase the efficiency of converting −COOH to triketone groups. Another solution of DCC (8.27 g, 40 mmol) in DMSO (50 mL) was prepared and added to the aforementioned solution stepwise. After reacting at room temperature for 48 h, the precipitate was removed with filtration. The filtrate was added to 1.0 N HCl_(aq)_ (1 L). The precipitate was collected by filtration and then dissolved in ethyl acetate (EA, 200 mL). The solution was extracted with 1.0 M NaOH_(aq)_ (100 mL) 3 times. The collected aqueous phase was extracted with EA for 3 times. The EA solution was collected. The solvent was removed with a rotary evaporator. The resulting powder was washed with water using a Soxhlet extractor and then dried at 70 °C under a vacuum. The product of OL-TK was obtained with a yield of about 49%.

### Synthesis of OLTK-CR-2

TREN (0.068 mL) and OL-TK (0.25 g, 0.68 mmol) were dissolved in DMSO (2.0 mL). The equivalent ratio of the primary amine to the triketone groups in the reaction mixture was 2.0. The solution was poured into a silicone mold. The sample was thermally treated at 60 °C for 3 h, 90 °C for 3 h, and 120 °C for 18 h under a vacuum. The sample of OLTK-CR-2 was obtained. Other samples with different X values were prepared in the same manner.

### Synthesis of DKAV-L-*X* Samples

For the preparation of DKAV-L-3, FTK (2.2 g, 5.5 mmol) was dissolved in a DMAc aqueous solution (90 vol %). OLTK (64.6 mg) and TREN (0.588 mL) were added to the solution. After being stirred at 60 °C for 5 min, the solution was poured into an aluminum mold and thermally treated at 60 °C for 2 h and 170 °C for 1 h. Post-thermal treatment on the sample was conducted with thermal pressing at 190 °C and 14 MPa for 0.5 h.

### Thermal Recycling of DKAV-L-*X* Samples

DKAV-L-3 was ground into a powder. The collected powders were collected in a stainless mold and thermally pressed at 190 °C and 14 MPa for 20 min. The recycled sample (DKAV-L-3/R1) was obtained. Other samples were recycled in the same manner.

## Supplementary Material



## Notes

More figures (Figures S1 to S9) mentioned and discussed in the main text (PDF)
