# Supplementary material for: Triketone-Modified Lignin and the Corresponding Biomass-Based Diketoenamine Resins: Synthesis and Properties
Source: ACS Omega. 2026 Jan 19;11(4):5261–70. doi: 10.1021/acsomega.5c07915 (PMC12878709; doi:10.1021/acsomega.5c07915)
Supplement: Supplementary file 1 [file ao5c07915_si_001.pdf]

# Supporting Information (SI) for

## Triketone-modified lignin and the corresponding biomass-based diketoenamine resins: synthesis and properties

Nien-Hsun Wu[†], Yi-Ming Sun and Ying-Ling Liu[†*]

† Department of Chemical Engineering, National Tsing Hua University, No. 101, Sec. 2, Kuang-Fu Road, Hsinchu 300044, Taiwan

Department of Chemical Engineering and Materials Science, Yuan Ze University, Chungli, Taoyuan 320315, Taiwan

* Corresponding author, E-mail: liuyl@mx.nthu.edu.tw

**6 Pages, 8 Figures**

**Table of Content** **Page**

***Figure S1.*** *(a) FTIR spectrum and (b) $^1$H NMR spectra of WSOL and OL-TK.* ........................................................................................................ **S2**

***Figure S2. Figure S2.*** *FTIR spectra recorded on OL-TK and OLTK-CR-2.* ........................................................................................................ **S3**

***Figure S3.*** *(a) photographs showing the thermal recycling on OLTK-CR-2 resin; (b) cross-sectional SEM micrograph recorded on thermally recyclec OLTK-CR-2 (OLTK-CR2/R1).* ...................................................................... **S3**

***Figure S4.*** *The acid-catalyzed aminolysis reaction and enamine–enol tautomerism of diketoenamine.* ................................................................. **S4**

***Figure S5.*** *Chemical structure of the furan-triketone compound (FTK) reported in the previous publication (Reference 22 in the main text).* ........................................................................................................ **S4**

***Figure S6.*** *Cross-sectional SEM micrographs (a) DKAV-L-0, (b) DKAV-L-3, (c) DKAV-L-7 and (d) DKAV-L-10.* ........................................................ **S5**

***Figure S7.*** *Strain sweep measurements on the DKAV-L-Y samples at 180 $^o$C with a frequency of 1.0 Hz and an applied force of 0.01 N.* ........................................................................................................ **S6**

***Figure S8.*** *Stress relaxation curves recorded on (a) DKAV-L-0, (b) DKAV-L-3, (c) DKAV-L-7, and (d) DKAV-L-10 samples at different temperatures.* .................................................................................. **S6**

***Figure S9.*** *Photographs showing the physical recycling and reprocessing properties of the DKAV-L-0, DKAV-L-3, and DKAV-L-7 samples.* ........................................................................................... **S7**

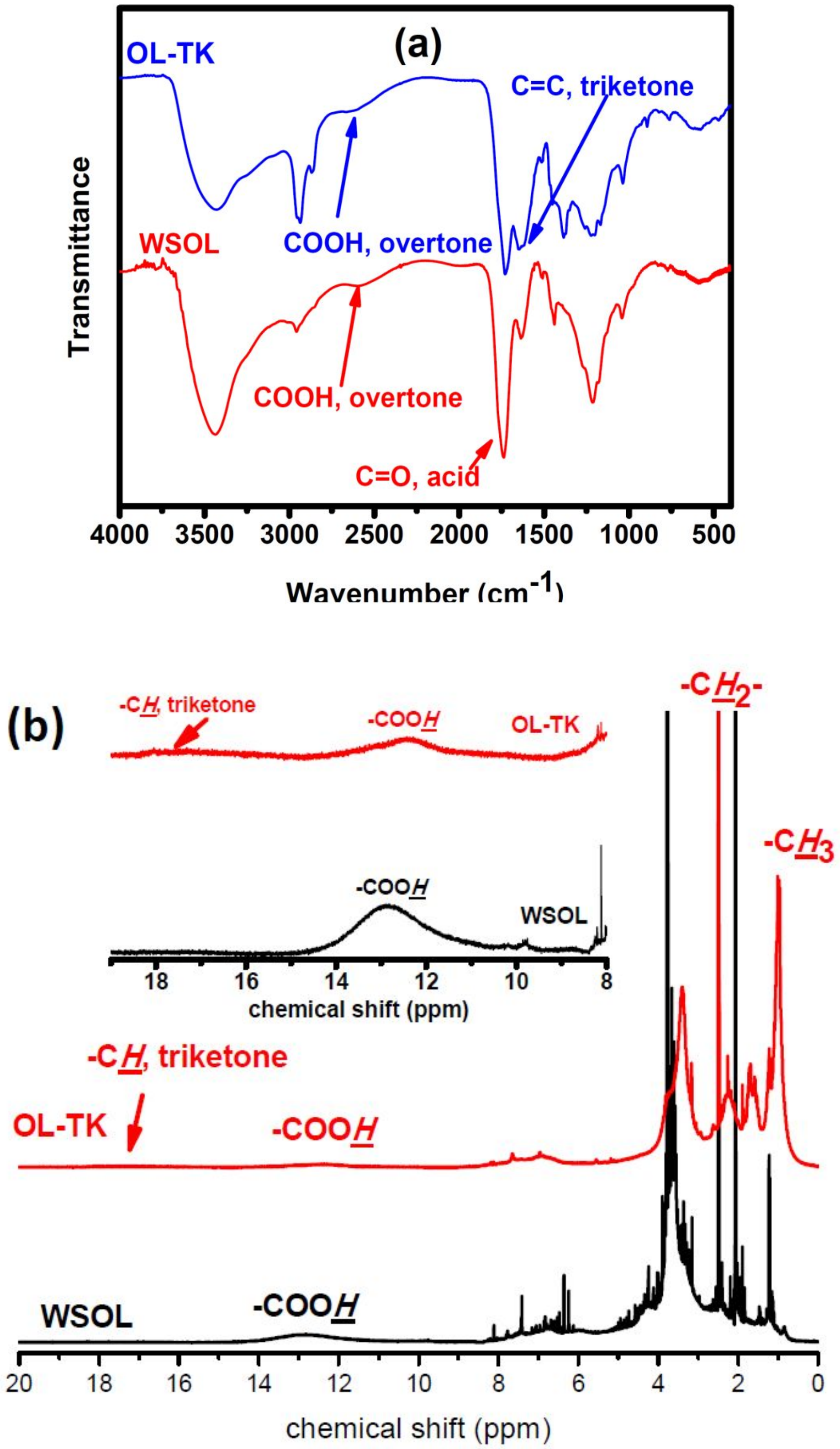


***Figure S1***. *(a) FTIR spectrum and (b)* $^{1}$*H NMR spectra of WSOL and OL-TK.*

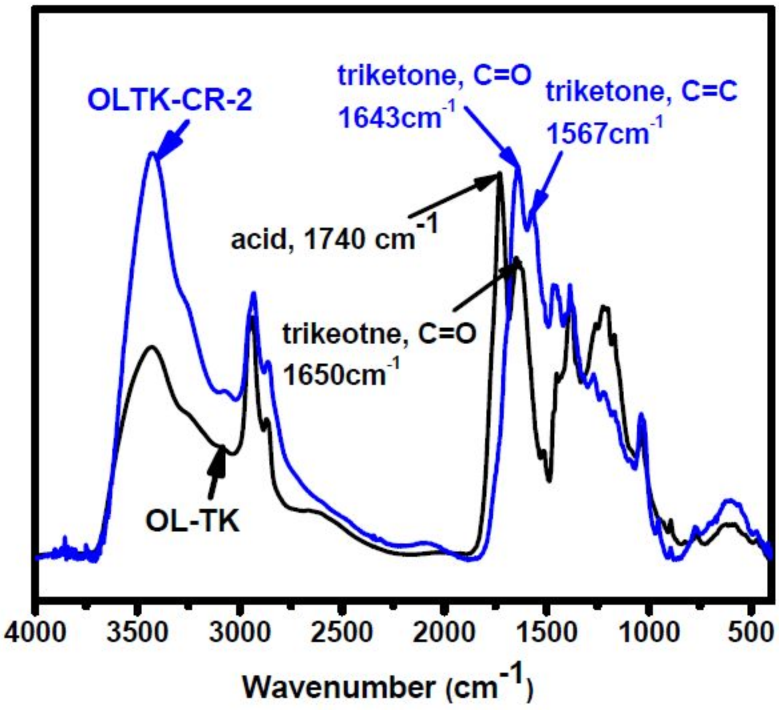


***Figure S2.*** *FTIR spectra recorded on OL-TK and OLTK-CR-2.*

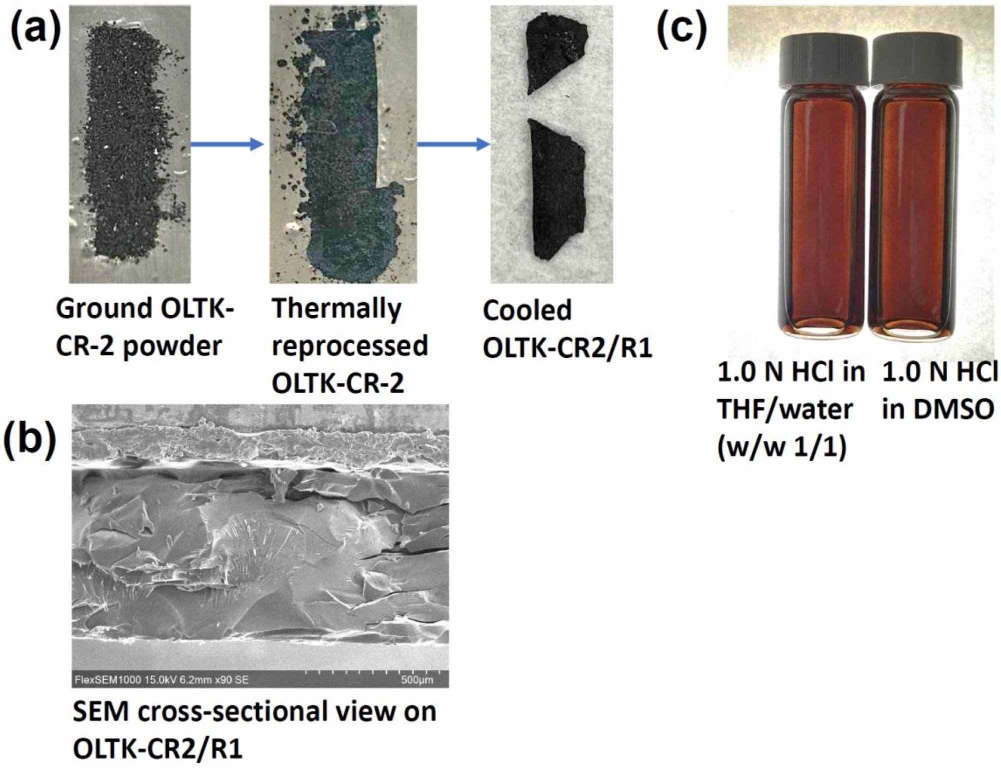


***Figure S3.*** (a) photographs showing the thermal recycling on OLTK-CR-2 resin; (b) cross-sectional SEM micrograph recorded on thermally recycled OLTK-CR-2 (OLTK-CR2/R1).

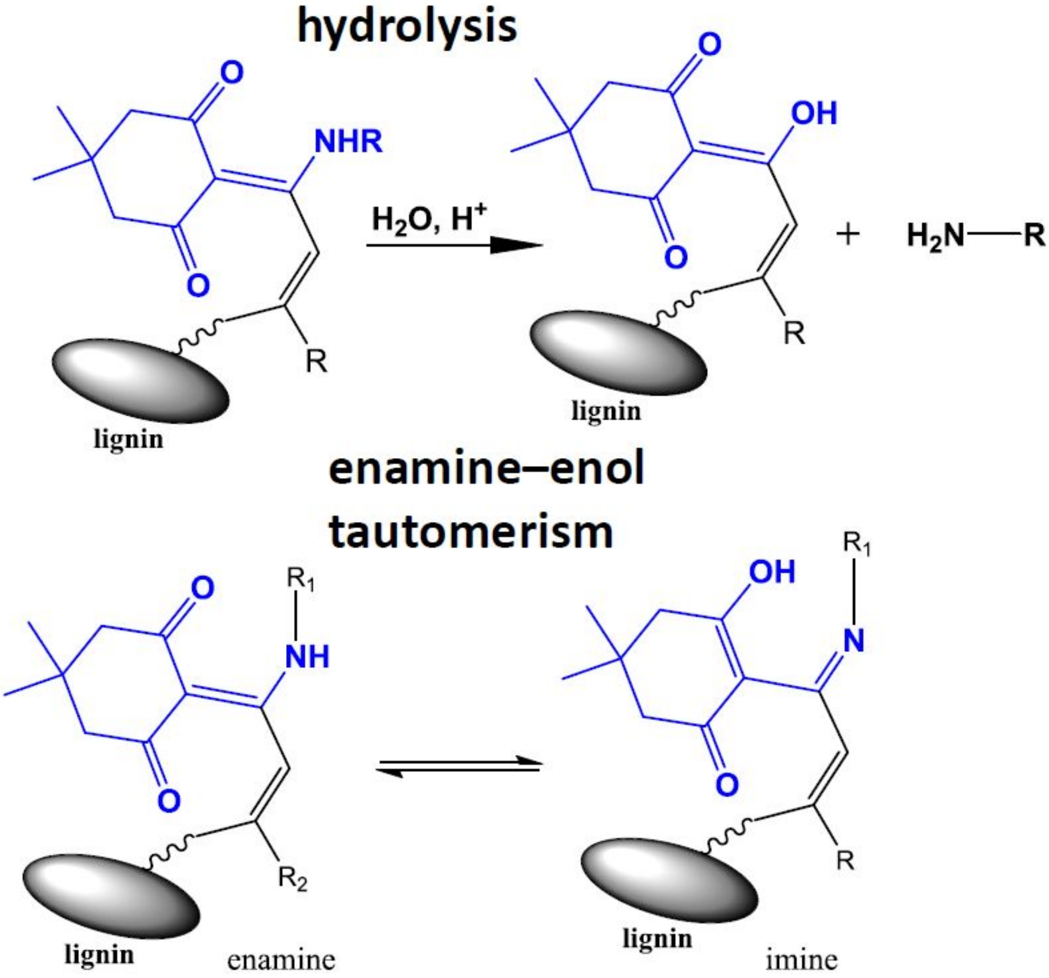


***Figure S4.*** *The acid-catalyzed hydrolysis reaction and enamine–enol tautomerism of diketoenamine.*

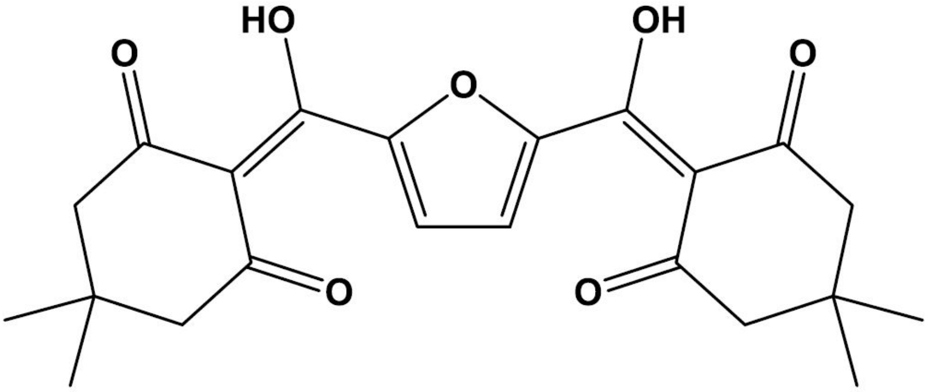


***Figure S5.*** *Chemical structure of the furan-triketone compound (FTK) reported in the previous publication (Reference 22 in the main text).*

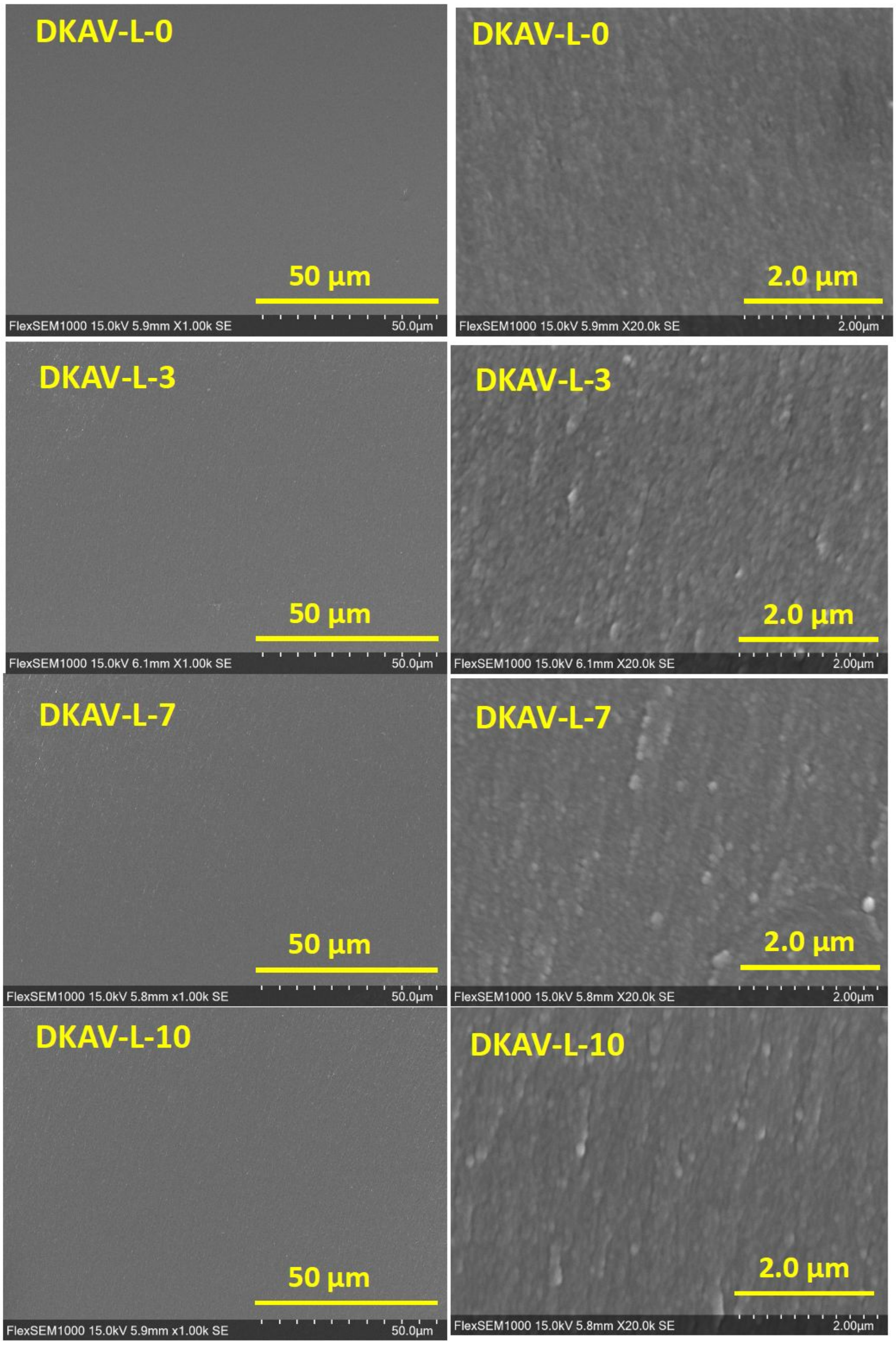


**Figure S6**. Cross-sectional SEM micrographs (a) DKAV-L-0, (b) DKAV-L-3, (c) DKAV-L-7 and (d) DKAV-L-10.

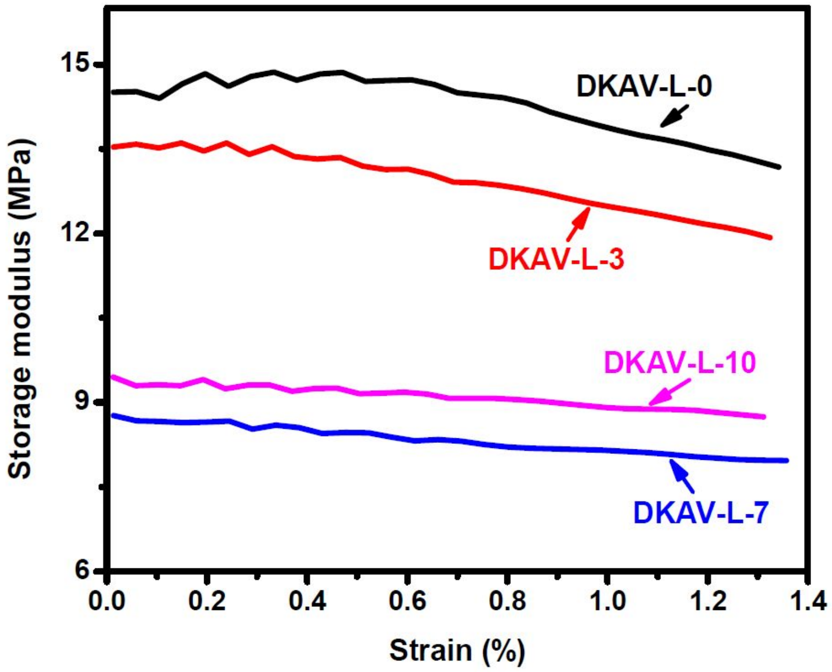


***Figure S7.*** *Strain sweep measurements on the DKAV-L-Y samples at 180 °C with a frequency of 1.0 Hz and an applied force of 0.01 N.*

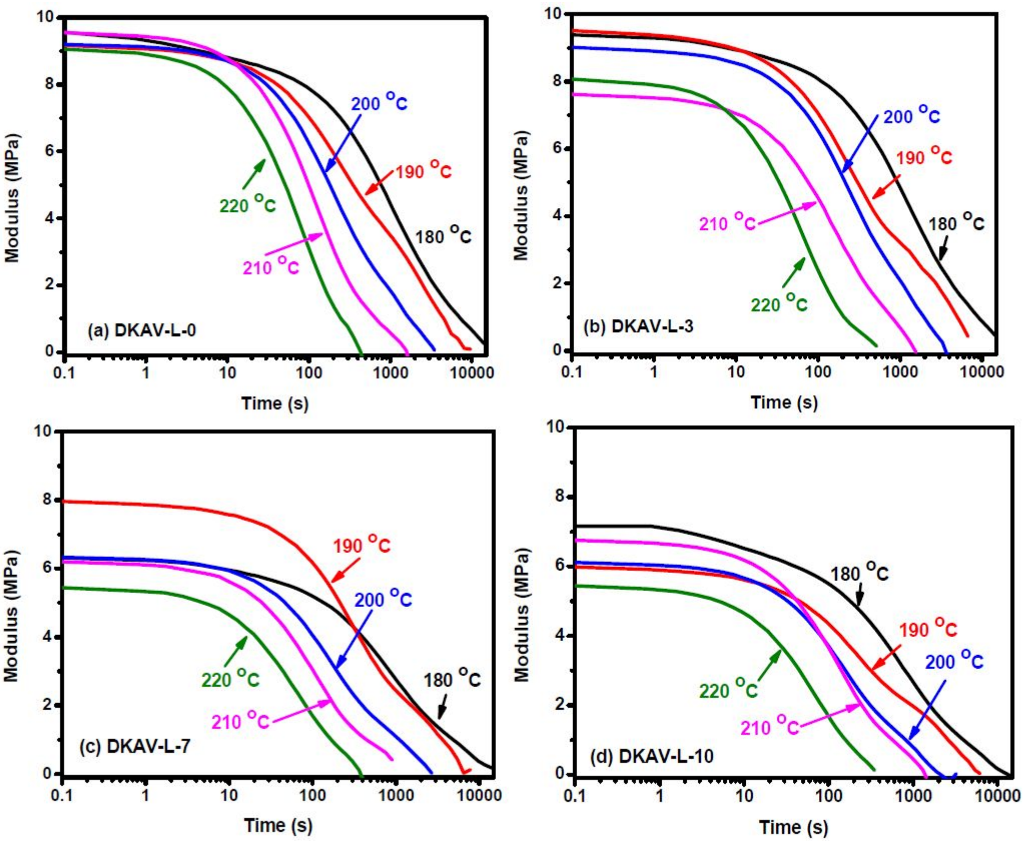


***Figure S8.*** *Stress relaxation curves recorded on (a) DKAV-L-0, (b) DKAV-L-3, (c) DKAV-L-7, and (d) DKAV-L-10 samples at different temperatures.*

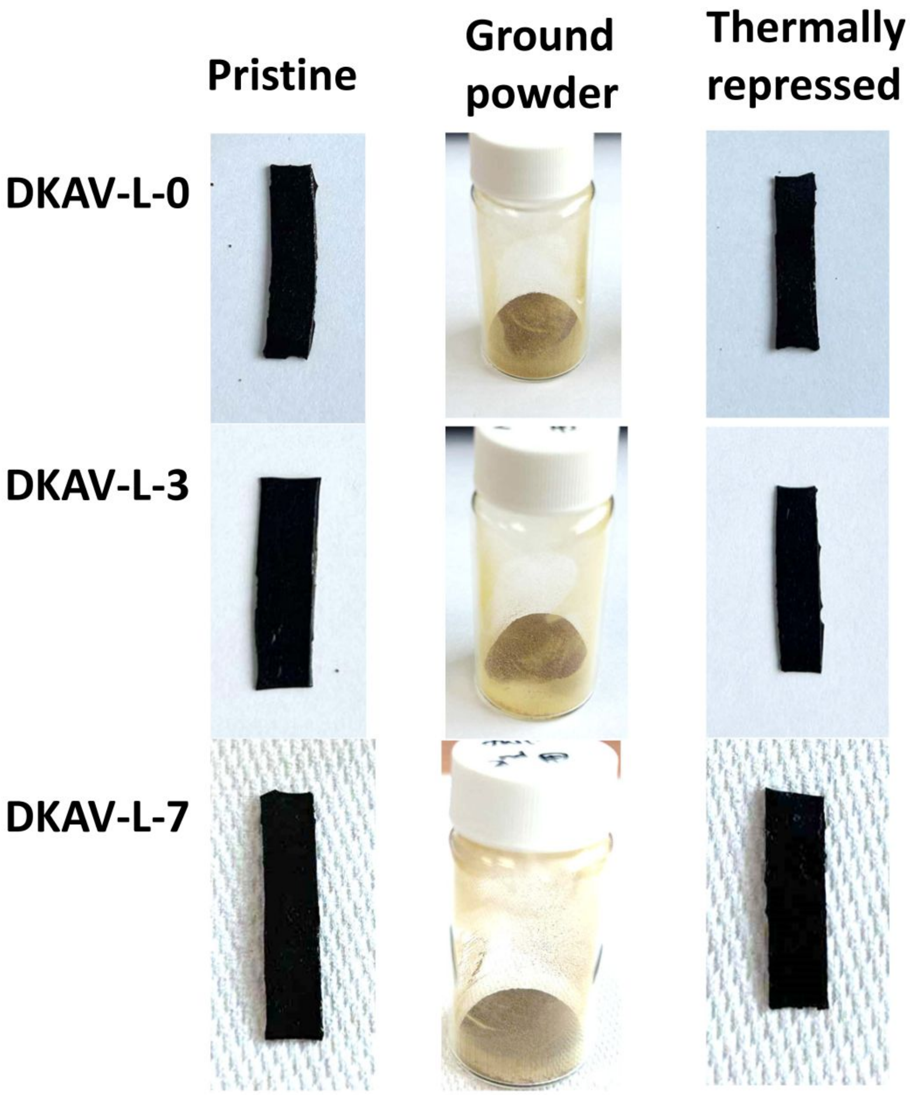


***Figure S9.*** *Photographs showing the physical recycling and reprocessing properties of the* DKAV-L-0, *DKAV-L-3, and DKAV-L-7 samples.*
